# Role of hypoxia in the tumor microenvironment and targeted therapy

**DOI:** 10.3389/fonc.2022.961637

**Published:** 2022-09-23

**Authors:** Gaoqi Chen, Kaiwen Wu, Hao Li, Demeng Xia, Tianlin He

**Affiliations:** ^1^ Department of Hepatobiliary Pancreatic Surgery, Changhai Hospital, Second Military Medical University (Naval Medical University), Shanghai, China; ^2^ Department of Gastroenterology, The Third People’s Hospital of Chengdu, The Affiliated Hospital of Southwest Jiaotong University, Chengdu, China; ^3^ Deparment of Neurology, Affiliated Hospital of Jiangsu University, Jiang Su University, Zhenjiang, China; ^4^ Luodian Clinical Drug Research Center, Shanghai Baoshan Luodian Hospital, Shanghai University, Shanghai, China

**Keywords:** Tumor microenvironment, immunity, metabolism, hypoxia, targeted therapy

## Abstract

Tumor microenvironment (TME), which is characterized by hypoxia, widely exists in solid tumors. As a current research hotspot in the TME, hypoxia is expected to become a key element to break through the bottleneck of tumor treatment. More and more research results show that a variety of biological behaviors of tumor cells are affected by many factors in TME which are closely related to hypoxia. In order to inhibiting the immune response in TME, hypoxia plays an important role in tumor cell metabolism and anti-apoptosis. Therefore, exploring the molecular mechanism of hypoxia mediated malignant tumor behavior and therapeutic targets is expected to provide new ideas for anti-tumor therapy. In this review, we discussed the effects of hypoxia on tumor behavior and its interaction with TME from the perspectives of immune cells, cell metabolism, oxidative stress and hypoxia inducible factor (HIF), and listed the therapeutic targets or signal pathways found so far. Finally, we summarize the current therapies targeting hypoxia, such as glycolysis inhibitors, anti-angiogenesis drugs, HIF inhibitors, hypoxia-activated prodrugs, and hyperbaric medicine.

## Introduction

A recent study shows that the global cancer burden is increasing (1). The research on the molecular mechanism and target of the occurrence and development of malignant tumors may be a breakthrough to solve this problem. Recently, it has been proved that TME is a key factor in the occurrence and development of malignant tumors, which is being the well-known hotspot. However, due to the influence of tumor cells and abnormal vascular structure, TME often shows the characteristics of hypoxia, especially in solid tumors.

Under the condition of hypoxia, the expression of HIF increases, and a series of changes have taken place in the metabolic mode and immune function of TME. In order to adapt to the influence of hypoxia, tumor cells have changed their metabolic mode and obtained energy through glycolysis. Meanwhile, immune cells are regulated by hypoxia and have different effects. Among them, the function of immune cells that play an anti-tumor role is inhibited, such as cytotoxic T cells, B cells, and natural killer (NK) cells. However, the expression of immunosuppressive cells such as marrow-derived suppressor cells (MDSC) and regulatory (Treg) T cells is up-regulated. The changes of metabolism and immune effect provide an excellent living environment for tumor cells and hinder the effect of anti-tumor treatment. In addition, while providing survival conditions for tumor cells, TME under hypoxia obstructs the effect of antitumor drugs by hindering drug delivery (2–4). Therefore, traditional chemotherapy and single dose immunotherapy cannot achieve satisfactory results, which makes the treatment of malignant tumors challenging (5, 6).

In conclusion, hypoxia, as an independent prognostic indicator related to poor survival rate of cancer patients, is expected to become an effective target for fighting tumor and alleviating drug resistance (7). After summarizing, we found that people are increasingly interested in the field of hypoxia in TME, and hundreds of relevant academic papers in this field have been published (8). Among the published studies, research targeting metabolic enzymes, HIF, and angiogenesis related factors have made breakthroughs to varying degrees. Currently, what’s exciting is more than 500 clinical trials have been adopt. In this review, we describe the effects of hypoxia on the proliferation, metastasis, and drug resistance of tumor cells in the TME from the perspectives of immunity, metabolism and HIF, and summarize the different treatment strategies targeting hypoxia. Finally, we summarized the current measures to combat drug resistance and the prospects for future research in this field.

## Effects of hypoxia on TME

**Figure 1:**
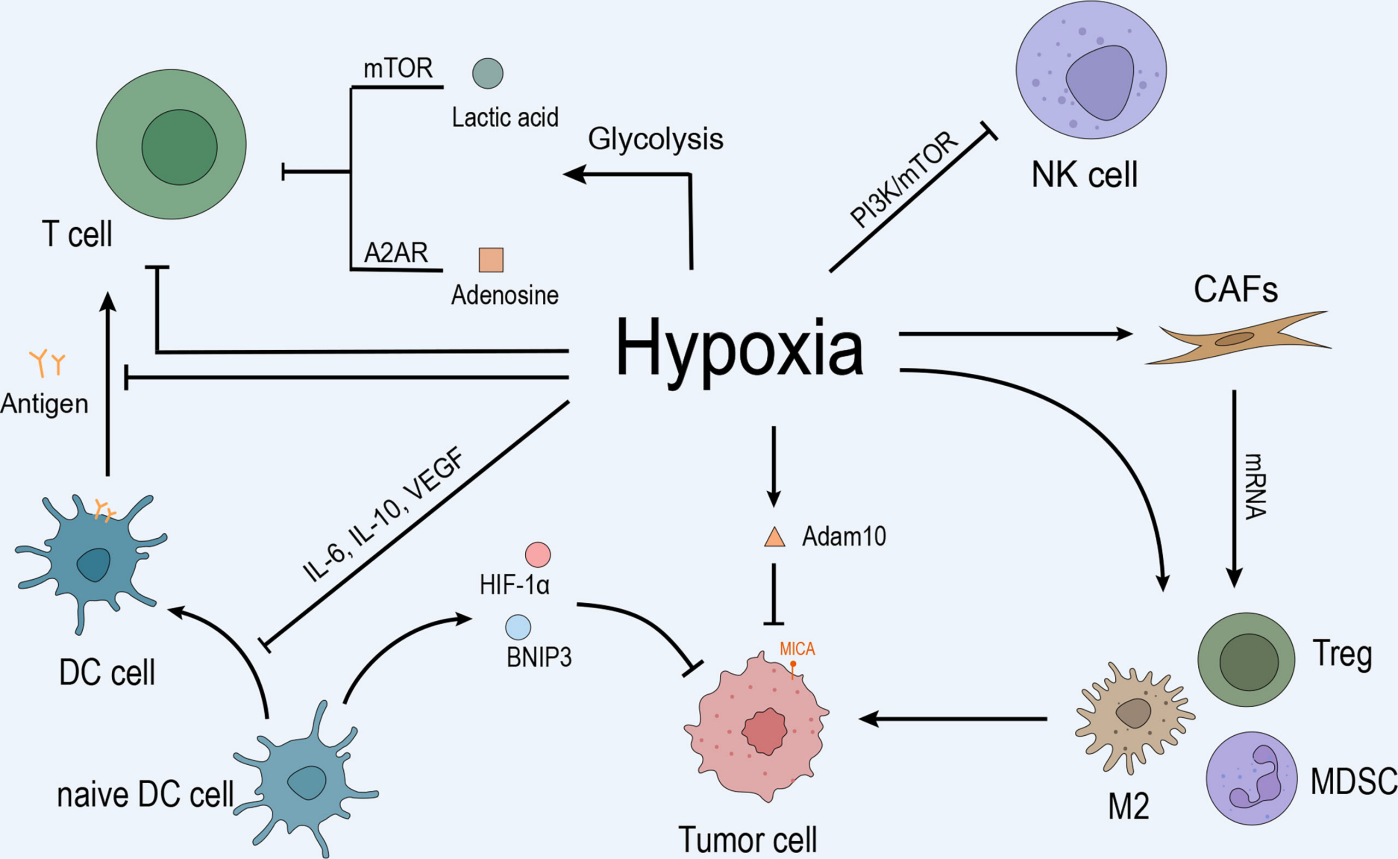
Hypoxia inhibits the immune response by inhibiting immune cells, recruiting immunosuppressive cells, regulating CAFs, promoting tumor cell growth, and mediating immune escape. **(A)** Anoxic metabolites, lactic acid, and adenosine inhibit T cell effector function and proliferation by blocking the mTOR pathway and interacting with the A2A receptor on the T cell surface. Hypoxia promotes T cell apoptosis and directly inhibits T cell proliferation and differentiation. Hypoxia upregulates IL-10, VEGF, and other cytokines through HIF-1α and inhibits the differentiation and maturation of DCs, leading to the inhibition of T cell function. Moreover, hypoxia-induced high levels of HIF-1α and BNIP3 promote programmed cell death in tumor cells captured by DC. In addition, hypoxia inhibits NK cell function by activating the PI3K/mTOR signaling pathway. **(B)** Hypoxia induced the mRNA expression of *TGF-β*, *VEGF*, *IL-6*, *IL-10*, and *PD-L1* and promoted CAF participation in the recruitment of MDSCs, Tregs, and type 2 TAMs to maintain the immunosuppressive state of the microenvironment, promoting tumor cells to evade immune surveillance. **(C)** Hypoxia upregulates the expression of MMP adam10 and induces the immune escape of tumor cells.

TME is a cellular environment that harbors the tumor, composed of tumor cells, fibroblasts, immune cells (T cells, B cells, natural killer (NK) cells, and tumor-associated macrophages (TAMs)), blood vessels, signaling molecules, and extracellular matrix (9, 10). Hypoxia is ubiquitous property in the TME, especially in solid tumors. Abnormal blood vessels in the tumor tissue cannot meet the excessive oxygen and nutrient demand for its rapid growth, leading to uneven hypoxia. Thus, the area away from the blood vessels was anoxic, while the adjacent tumor tissue was hyper-oxygenated. A recent study suggested that hypoxia affects the immune microenvironment and makes tumor cells escape from immune monitoring and killing (11). As shown in **Figure 1**, the anoxic area in tumor tissue hinders the infiltration of immune cells and promotes the growth of tumor cells (12).

### Hypoxia inhibits the function of immune cells

Effector T cells are the main components of immune response in the tumor immune microenvironment, for example, the proliferation and differentiation of T cells determine the strength of the antitumor immune response. Several studies have confirmed that hypoxia is a major regulatory factor that inhibits the function and proliferation of T cells (11, 13). A2A receptor (A2AR) is a kind of G protein coupled receptor with high affinity for adenosine, which is expressed on T cells, NK cells, macrophages, and other immune cells (14). Tumor cells can inhibit the response of immune cells through adenosine-a2ar pathway and promote tumor cells to escape immune surveillance (14). Under hypoxic conditions, tumor cells exploit the glycolytic process to accumulate metabolites, such as lactic acid and adenosine, in the TME. The accumulation of lactic acid and adenosine inhibits T cell effector function and proliferation by blocking the Sirolimus pathway and interacting with the A2AR on the T cell surface (15, 16). On the other hand, hypoxia promotes the apoptosis of T lymphocytes, delays the differentiation of effector cells, and reduces the production of effector T cells and interferon-gamma (IFN-γ) (17). In order to inhibit T cell proliferation, differentiation, and other functional cells, such as dendritic cells (DCs) that present antigens to T cells and activate the Hapten response, which also affected by hypoxia.

B cells, as the main carrier of humoral immunity, play a key role in the production of antibodies. Therefore, the functional defect of B cells will lead to the decline of immune effect. In the hypoxic tumor microenvironment, the transcription and metabolism of B cells are mainly affected by hypoxia inducible factor-alpha (HIF-1α) and myelocytomatosis virus oncogene cellular homolog (MYC) (18, 19). Myc gene specifically regulates the growth and metabolism of these various types of cells and has the potential to cause cancer (19, 20). To meet the energy demand of proliferation, B cells with malignant tendency show high metabolic behavior different from normal cells and show a vicious cycle. However, this differential performance still needs further research, and may become a treatment strategy in the future (19).

DCs are immune cells that capture and present antigens through major histocompatibility complex (MHC) glycoproteins. Many studies have shown that interleukin-6 (IL-6), IL-10, vascular endothelial growth factor (VEGF), and the other cytokines are upregulated by hypoxia, which inhibits the differentiation and maturation of DCs and T cell function (21, 22). *BCL2* gene, as a key gene regulating apoptosis, is up-regulated in tumor cells. The BCL2 encoded protein can achieve programmed cell death by regulating proteolytic caspase activation (23, 24). Immature DCs express high levels of HIF-1α and upregulated BCL2 and adenovirus E1B19 kDa interacting protein 3 (BNIP3), inducing programmed cell death in captured cells (25). Yang et al. found that the phagocytic capacity of DCs was lower than normal under hypoxia (26). In addition, hypoxia-stimulated DCs induce the differentiation of naive T cells into the Th2 phenotype, which in turn inhibits T cell proliferation (27). Hypoxia affects the function and differentiation ability of DCs, indicating that the activation ability of DCs to T cells. After that, the effect of T cell immunity on tumor cells is reduced, promoting the immune escape of tumor cells (26, 28).

NK cells constitute a class of naturally occurring cytotoxic lymphocytes. The ability of NK cells to kill tumor cells is inhibited under hypoxia (29) *via* the activated phosphatidylinositol 3 kinase (PI3K)-mTOR signaling pathway. In addition, hypoxia decreases the expression of the tumor cell surface recognition molecule MICA by upregulating the expression of metalloproteinase 10 (MMP10), thus downregulating the expression of NK and Natural killer group 2 member D (NKG2D) on T cells and inducing the immune escape of tumor cells (30). NKG2D is an activated receptor of immune cells such as T cells and NK cells, which could turn on the immune effect function. The upregulation of its ligand MCIA/MCIB on the surface of tumor cells is conducive to the continuous development of anti-tumor immunity (31). Therefore, the NKG2D ligand (NKG2DL) as a therapeutic target has become a research hotspot in recent years, in which the research progress in the fields of tumor vaccines has made exciting results (32, 33).

Immune checkpoint refers to the ligand-receptor pairs that stimulate or inhibit the immune response, which also affected by hypoxia (34). Hypoxia modulates PD-L1, human leukocyte antigen g (HLA-G), CD47, and the immune checkpoint V-domain IG suppressor of T cell activation (VISTA) to form an inhibitory immune microenvironment, promoting immune escape of tumor cells. PD-1 is widely distributed on the surface of lymphocytes. Under hypoxic conditions, the level of PD-L1 protein on the tumor cell surface is enhanced, and it combines with the PD-1 receptor on the activated T cell surface to produce the immunosuppressive effect (28, 35). Presently, antibodies against PD-1 and PD-L1 have achieved preliminary success in the clinic (36). Human leukocyte antigen G (HLA-G) is another checkpoint molecule involved in tumor immune escape and is strongly associated with increased tumor invasiveness and suppression of immune cell function (37, 38). In addition, the immune checkpoint molecules involved in the inhibition of T cell proliferation and activity under hypoxia conditions include VISTA (11).

### Hypoxia modulates immunosuppression

Cancer-associated fibroblasts (CAFs) are similar to inflammation-associated fibroblasts, and participate in tumor cell progression and immune cell regulation during the antitumor immune response (39). Ziani et al. demonstrated that the mRNA expression of CAF-related immunosuppressive modulators, such as tumor growth factor-beta (TGF-β), VEGF, IL6, IL10, and PD-L1 increased significantly under hypoxia (40). CAFs are involved in the collection and differentiation of marrow-derived suppressor cells (MDSCs), regulatory T cells (Tregs), and type 2 TAMs in TME (41, 42). In addition to the recruitment of immunosuppressive cells, CAFs inhibit T cell immune response and enhance the tumor cell immunosuppressive response in the TME, which might be related to the inhibition of CAF, DC, and NK cell functions (43, 44). Under hypoxia, CD4^+^ T cells differentiate into Tregs by promoting Foxp3 transcription. Tregs are a subset of CD4 T cells and contribute to immunosuppression and tumor tolerance by producing TGF-β and suppressive effector T cells (21). MDSCs are immature myeloid cells that directly inihibit T cells, NK cells, and dendritic cells and promote angiogenesis in tumor tissue (45, 46). Chiu et al. demonstrated that under the influence of hypoxia, the differentiation of MDSCs was inhibited, but its immunosuppressive function was maintained (47). TAM is a major component of the solid TME (48). The two phenotypes of TAMs are M1-like and M2-like phenotypes (48). Type M2 TAMs are detected in anoxic regions and associated with immunosuppression, angiogenesis, tumor cell activation, and metastasis (49). Another study showed that prostaglandin E2, TGF-β, VEGF, IL-4, IL-6, and reactive oxygen species (ROS) were the major factors that induced TAMs to M2-type TAMs (29). In addition, hypoxia-mediated lactic acid accumulation under HIF-1α regulation increases the expression of VEGF and M2-like polarization of TAMs to maintain the immunosuppressive status of the TME (47, 50).

## Changes in tumor metabolism caused by hypoxia

**Figure 2:**
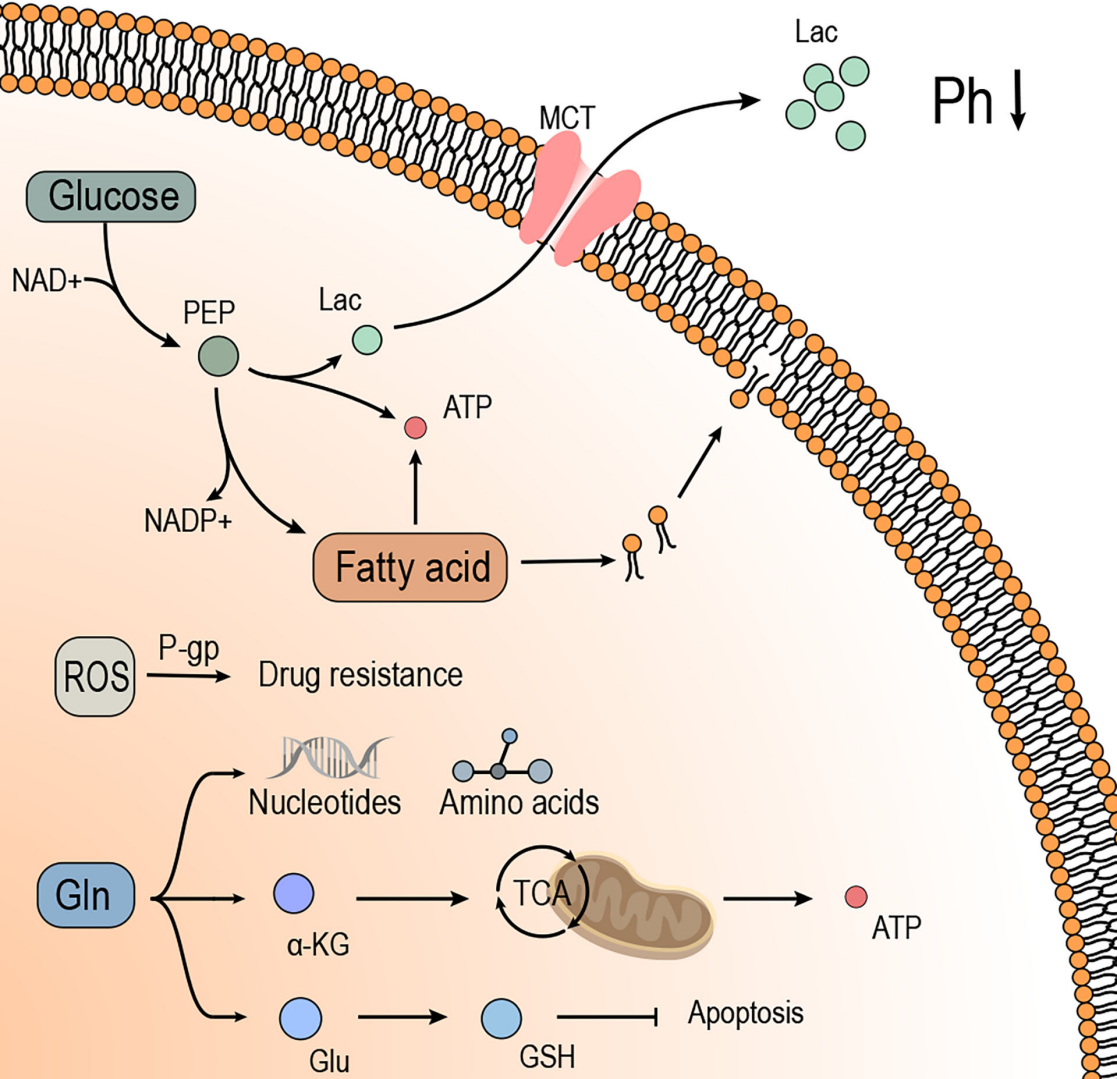
Hypoxia induces metabolic reprogramming of tumor cells, which provides energy and substrates for tumor cell growth and promotes drug resistance. I. Glucose provides energy to the tumor cells in the form of glycolysis, of which the metabolite Lac is transported to the outside of tumor cells through MCT, effectuating low pH and suppressing the immune effects. The intermediate products in glycolysis contribute to the synthesis of fatty acids and promote the growth and proliferation of tumor cells. II. Gln is broken down into a-KG in tumor cells to provide energy through the TCA cycle or raw materials to synthesize amino acids and nucleic acids in tumor cells. In addition, GLN expresses antioxidant ability by synthesizing GSH, which promotes drug resistance and anti-apoptosis in tumor cells. III. Fatty acids provide materials for the synthesis of biomembranes to meet the growth needs of tumor cells. The synthesis of fatty acids consumes PEP, which relieves the build-up of Lac from glycolysis. The breakdown of fatty acids produces large amounts of ATP, which provides energy for the growth and proliferation of tumor cells. IV. ROS induces drug resistance in tumor cells, associated with the P-gp.

Hypoxia affects the TME and alters the tumor and the surrounding tissue metabolism. With the progress of TME studies, metabolic reprogramming has been recognized as a hallmark behavior of malignant tumors. As shown in **Figure 2**, metabolic reprogramming is the metabolic modification of tumor cells to maintain growth and resist treatment. This reprogramming includes aerobic glycolysis and L-glutamine metabolism et al (51). Thus, it could be deduced that hypoxia inhibits the apoptosis of tumor cells by promoting the metabolic reprogramming of tumor cells.

### Glucose metabolism

Under aerobic conditions, cells produce pyruvic acid through the glycolytic process, which is oxidized in the mitochondria to produce energy. Under anoxic conditions, the energy of normal cells is mainly provided by glycolysis. However, most tumor cells tend to produce energy by glycolysis even under aerobic conditions. This phenomenon is known as the “Warburg effect” (52, 53). Through this phenomenon, tumor cells use glycolysis for energy and produce lactic acid. The lactic acid accumulates outside the tumor cell *via* activated monocarboxylic acid transporters, causing a low pH in the extracellular matrix. Some studies have shown that a low pH environment enhances the invasiveness of tumor cells and inhibits the cytotoxicity and proliferation of lymphocytes, which inhibits the functioning of immune effector cells in the TME (54). Nonetheless, as the acidic environment is corrected, the T cell effector function is restored (55). In addition, tumor cells use glycolytic metabolic intermediates to synthesize fats and proteins. The metabolic way of aerobic glycolysis weans the tumor cells off oxygen dependence, which is beneficial to the growth and proliferation of tumor cells in a hypoxic environment (56). Also, the multidrug resistance of tumor cells is closely related to the reprogramming of glucose metabolism. A current study suggested that this process is influenced by a combination of mechanisms, including “ion capture,” decreased apoptotic potential, gene changes (such as p53 mutation), and increased activity of P-gp, a multidrug transporter.

### Glutamine metabolism

Except for glycolysis, cancer cells under hypoxic conditions tend to choose an alternative substrate for energy metabolism, such as L-glutamine. Some studies have shown the critical role of L-glutamine plays in tumor cell proliferation as an alternative energy source for tumor metabolism (57). L-Glutamine is synthesized as glutamate, which is then converted into α-ketoglutaric acid through transamination and into the tricarboxylic acid cycle for energy metabolism to compensate for the reduced energy production from glycolysis (58, 59). In addition, glutamate provides nitrogen and carbon sources for tumor cells and participates in the synthesis of amino acids and nucleotides, which promotes the development of malignant tumors (60). On the other hand, L-glutamine could be used to synthesize glutathione, a crucial antioxidant that maintains the redox balance and prevents oxidative damage to cells (61, 62). Friesen et al. suggested that glutathione, a metabolite of L-glutamine, is involved in mediating drug resistance and anti-apoptosis in cancer cells which may be related to the antioxidant capacity of glutathione. Strikingly, when glutathione levels are decreased, drug and apoptosis resistance of tumor cells is restored (63).

### Fatty acid metabolism

The synthesis of biomembranes and signaling molecules is essential for the rapid proliferation and growth of tumor cells. Fatty acid is a critical raw material. Therefore, tumor cells have high levels of fatty acid synthesis. On the other hand, the synthesis consumes pyruvic acid, which slows the synthesis of lactic acid and prevents the excessive build-up of lactic acid. In addition, the decomposition of fatty acids provides energy for tumor cells and the free fatty acids of metabolic products act as signal molecules that activate various signaling pathways (64, 65). Hypoxia and fatty acid metabolism studies have shown that the occurrence of tumors is closely related to β-oxidation. The enzymes FASN, ACC, and ACLY involved in fatty acid metabolism are upregulated in tumors (66, 67). Another study showed that the efficacy of immunotherapy, T cell longevity, and antitumor effects are also affected by lipid metabolism (68).

## The role of HIF

HIF is a major factor that mediates tumor cells to adapt to hypoxia (69). HIF-1α transcription factor directly targets *VEGF*, *TGF-β*, *IL-10*, and *PD-L1* genes and regulates the tumor immunosuppressive response to CAFs (70–72). HIF is a heterodimer helical-loop protein consisting of an O_2_-sensitive α-subunit (including HIF-1α, HIF-2α, and HIF-3α) and a constitutive β-subunit (16). HIF-1α plays a key role in several steps of hypoxia induction (73). In case of hypoxia, HIF-1α breakdown is reduced and transferred to the nucleus when the function of prolyl hydroxylase (PHD-RRB is inhibited. In the cell nucleus, HIF-1α binds to HIF-1β to form heterodimers. HIF-1α/1β heterodimer activates the HIF target gene and promotes HIF expression by combining the HIF-1α/1β heterodimer with p300/CBP and hypoxia response element (HRE), thus regulating various biological processes of tumor cells, including metabolic reprogramming, immunoregulation, angiogenesis, tumor cell invasion, and drug resistance (74, 75).

Among them, HIF could inhibit the immune response by promoting the up regulation of immune checkpoints, apoptosis of cytotoxic T cells and blocking phagocytosis, which is conducive to the occurrence and development of tumor cells. Studies have reported that the up-regulated immune checkpoint PD-L1 in tumor cells presents HIF-1α dependence, promote the apoptosis of cytotoxic T cells, and participate in the immune escape of tumor cells (76, 77). Similarly, the expression of CD47 protein on the surface of tumor cells is also affected by HIF-1α, and hinder the phagocytic ability of phagocytes to tumor cells through phosphorylation of signal regulatory proteins on the surface of macrophages α (SIRP α) (78). In recent years, the research results of CD47 protein showed that the expression of CD47 protein also inhibited the function of cytotoxic T cells and NK cells. Therefore, a series of clinical studies targeting CD47 protein are expected to bring new hope to the field of tumor therapy (79, 80). In addition, vascular endothelial growth factor (VEGF) is up-regulated affected by HIF-1α, which could promote metastasis and immune escape by inducing tumor angiogenesis. It is believed that the disorder of TGF-β is related to the occurrence and development of tumors, and enhances the invasive ability of tumor cells by inducing epithelial to mesenchymal transition (EMT) (81, 82). The research of Huang et al. showed that the HIF-1α regulate the function of TGF-β by forming Smad-HIF-1α complex under hypoxia, and then regulate the progress of tumor cells (83).

In addition to the inhibition of immune effects in TME, HIF-1 activates or inhibits the genes of key proteins in glycolysis pathway to regulate the metabolic process of tumor cells in hypoxic environment. Proteins or genes involved in the regulation of glycolysis pathway and regulated by HIF-1 include those involved in encoding glucose transporters (GLUT1 and GLUT3), hexokinases (HK1 and hK2), lactate dehydrogenase A (LDHA), etc (84–88). At the same time, oxidative phosphorylation related genes and proteins are negatively regulated by HIF-1. The differential regulation of proteins or genes relating glycolysis and oxidative phosphorylation by HIF-1 is conducive to the adaptation of tumor cells to achieve glucose metabolism reorganization. In addition, tumor cells reprogrammed by glucose metabolism have higher “competitiveness” to glucose in the microenvironment, so T cell apoptosis is induced by inhibition of energy metabolism, which aggravates the inhibition of T cell function. Except the glycolysis, HIF, as a major regulator, is involved in regulating glutamine metabolism in tumor cells (89). Under the condition of hypoxia, HIF-2α causes the change in *SLC1A5* gene encoding neutral amino acid transporter, which mediates the reprogramming of glutamine metabolism and the resistance to gemcitabine in tumor cells (90). Moreover, HIF inhibit α- Ketoglutarate participates in the tricarboxylic acid cycle by promoting α- Ketoglutarate dehydrogenase (αKGDH) degradation (91). In addition, HIF is also involved in regulating many metabolic pathways, such as fatty acids, pentose phosphate and adenosine, so as to provide a metabolic basis for the progression and metastasis of tumor cells.

In summary, the important role of HIF in tumor progression and its potential mechanism have been widely concerned by researchers. Further research in this field in the future is expected to help us have a deeper understanding of hypoxic TME, and bring new hope to the research of tumor targeted therapy (92).

## Effect of hypoxia on tumor oxidative stress

ROS are the main molecules produced by oxidative stress and have been considered major factors in the tumor occurrence, development, and recurrence. The ROS in tumor cells originate from mitochondria (93). Under the influence of hypoxia, the oxygen utilization efficiency of tumor cells is decreased. Therefore, electron transport efficiency through the mitochondrial complexes in the electron transport chain (ETC) is reduced, resulting in abundant ROS in cells (94, 95). Notably, various concentrations of ROS exert different effects on tumor cell production (96). High concentrations of ROS disrupt the proteins and nucleic acids and induce apoptosis of tumor cells through oxidative stress (97). A low concentration of ROS can promote the development and metastasis of tumor cells (98). In addition, the concentration of ROS affects the sensitivity and resistance of tumor cells to chemotherapeutic drugs, which might be related to the level of p-glycoprotein (P-gp) in drug resistance (99) (**Figure 2**).

## Effect of hypoxia on drug resistance of tumor

To date, chemotherapy is the cornerstone of cancer treatment. Anoxic metabolic disorders and changes in the microenvironment severely inhibit the efficacy of drugs such as Bleomycin. This could be because the oxygen-dependent chemotherapy drugs are more active when oxygen is available (100), while hypoxia directly inhibits the antitumor function of oxygen-dependent chemotherapeutic drugs (101). In addition, hypoxia indirectly reduces the efficacy of the drugs by interfering with the cell cycle, promoting DNA repair, and reducing the sensitivity of p53-mediated apoptosis (29, 102). Intriguingly, immunotherapy has developed rapidly in the past decade and has produced significant clinical results. However, the current studies have suggested that hypoxic stimulation significantly limits the effectiveness of immunotherapy (21, 103, 104). About 33% of patients who responded to immunotherapy suffered resistance again (6). Hypoxia effectuates metabolic changes in tumor cells, including low pH, high ROS, abnormal blood vessels, and proliferating fibrous tissue. These changes are beneficial to tumor cell survival, provide anti-apoptosis advantages, inhibit drug penetration, and promote the development of cancer and drug resistance (105).

To confront the challenge of drug resistance, the main methods include early diagnosis, combined multi drug therapy, and adaptive therapy (106). Effective evaluation molecules could provide a good reference for the early diagnosis and progress of malignant tumors. Quantitative monitoring of circulating tumor DNA (ctDNA) is expected to become an important means of early diagnosis and dynamic monitoring of cancer, which will help to improve the overall survival rate and guide individualized treatment (107). Combined with positron emission tomography and computed tomography (PET-CT) imaging results, evaluate the curative effect. And then adjust the drugs, so as to avoid the emergence of drug resistance and achieve better curative effect (108). Meanwhile, precise drug delivery methods such as antibody drug conjugate (ADC) could increase the curative effect by increasing the local drug concentration (109). In addition, multi drug combination therapy is still an effective measure to combat drug resistance at present. The research of Niu et al. shows that the combined application of vibostolimab (anti-TIGIT humanized IgG1 monoclonal antibody) and pembrolizumab can significantly inhibit and improve the drug resistance of non-small cell lung cancer (NSCLC) (110).

## Strategies for targeting hypoxic microenvironments

Bibliometric research published on *Frontiers in oncology* shows that researchers’ interest in the research of tumor microenvironment is continuously rising from 2011 to 2021 (8). Moreover, the current research hotspot in this field focuses on the energy metabolism, oxidative phosphorylation, liposomes and other new drug delivery routes in TME (8). Therefore, in order to facilitate readers to understand the progress in this field, we summarize the treatment strategies that targeted at hypoxia. It is gratifying that several potential anti-tumor targets have been found (**Table 1**), including targeting hypoxia, glycolytic drugs, abnormal angiogenesis, and HIF drugs.

**Table 1:** Target drugs for metabolism, HIF, and other pathways.

Category	Pathway/Target	Drugs	Reference
HIF	HIF-1α/VEGF	PKM2, benzofuran, derivatives, BITC, VHH212, P-AscH, Alpha-solanine, TX-2098	(111–117)
	Others	USP25	(118)
Metabolism	Glycolysis	MIR210HG, UHRF1,2-deoxyglucose (2-DG),3-bromopyruvate (3-BP), UBR5, MTAP, CPI-613, ERO1L, BZW1	(119–127)
	Glutamine metabolism	CB-839 mTORC1, EGFR-Pak, SUCLA2, SLC1A5	(128–132)
	Pentose phosphate pathway	PRLR, p16, KRT6A	(133–135)
	Hexosamine biosynthesis pathway	GFAT1, PMG3, NAGK, NF-κB	(136–139)
	Branched chain amino acid (BCAA) metabolism	BCAT2, BCKDHA, BCAT1	(140–142)
	OXPHOS	UQCRC1, metformin, 64 (DX3-234), ONC212, Phenformin	(143–147)
Others	Autophagy	Hydroxychloroquine, BML-275, MEKINIST, SEMA3A	(148–151)
	Antiangiogenic agents	Sunitinib, ceritinib, EndoTAG-1, bevacizumab	(152–155)
	Hypoxia-activated prodrug	TH-302, Evofosfamide, YME1L, HMGCR inhibitors, SQLE	(155–160)

Summary of the current therapeutic pathway and targets related to hypoxia, such as HIF and metabolism, and the drugs corresponding to each approach and target.

The energy metabolism of tumor microenvironment is a current research hotspot, and researchers have carried out a series of studies with it. A current study showed that glycolytic inhibitors effectively kill tumor cells that are not sensitive to chemotherapy drugs, even when they were present in multiple drug resistance cells (161, 162). Hexokinase 2 (HK2) plays a critical role in regulating aerobic glycolysis in tumor cells and has become one of the main targets of tumor therapy. A previous study showed that HK2 inhibitor 3-bromopyruvic acid (3-BP) significantly inhibits the progression and proliferation of tumor cells in HK2- expressing colorectal cancer. Moreover, apoptosis of tumor cells was induced by the signaling pathway of mitochondrial apoptosis (163). In recent years, the emergence of chemical dynamic therapy (CDT) has provided a new solution for cancer treatment (164). Interestingly, the amount of glutathione in the tumor cells directly affects the efficacy of chemotherapy. Glycolytic inhibitors could reduce the tumor’s glycolytic process and enhance CDT selectivity to tumor cells, thereby exploiting metabolic differences to achieve the specific treatment for tumor cells (165). In addition, OXPHOS inhibitors, such as metformin, improve hypoxia in the microenvironment by inhibiting the mitochondrial complex I, which reduces oxygen consumption in cells and corrects the hypoxic TME. Besides, OXPHOS also inhibits the upregulation of cancer subtypes, such as ovarian cancer, prostate cancer, and thyroid cancer (166, 167).

Abnormal tumor blood vessels are major factors in the continuous hypoxia of TME that hinder drug delivery (168). In the hypoxic TME, angiogenesis-promoting cytokines, such as VEGF and TGF-β, impede the differentiation and maturation of endothelial cells in neovascularization. As a result, malformed and poorly permeable new blood vessels aggravate the anoxic state of the tumor, making it difficult to deliver drugs effectively to the tumor (169). Antiangiogenic drugs, such as anti-VEGF antibodies, correct the abnormal blood vessels and promote the normalization of tumor blood vessels, which in turn alleviates hypoxia and improves the efficacy of conventional antitumor drugs (170, 171). However, angiogenesis inhibitors alone do not receive ideal therapeutic results, which might be related to the complex mechanisms of angiogenesis compensation (172). Therefore, VEGF inhibitors need to be used in combination with chemotherapy or immunotherapy, which has achieved satisfactory results in solid tumors, such as ovarian and breast cancer (173, 174).

HIF activity is mainly dependent on HIF-1α and plays a critical role in the regulation of hypoxic TME. Presently, studies on targeting HIF-1α are being widely carried out. Targeting the HIF-1α signaling pathway is effective in the treatment of solid tumors, such as pancreatic cancer (175, 176). Ubiquitin carboxy-terminal hydrolase L 1(UCHL1) is a ubiquitin-free enzyme that stabilizes its α-subunit (HIF-1α). UCHL1 inhibitors promote the degradation of HIF-1α and inhibit the activity of its downstream genes. Li et al. showed that the inhibition of the UCHL1-HIF-1 pathway decreases the expression of malignant tumor-related factors and eliminates UCHL1-mediated tumor cell proliferation and metastasis (177). In addition, Nelson et al. found that in the hypoxic microenvironment of pancreatic cancer, downregulation of USP25 reduced the transcriptional activity of HIF-1α, leading to cell death in the hypoxic core of the tumor without normal tissue affected (118). In another mouse model of pancreatic cancer, Xu et al. demonstrated that the Benzofuran derivative inhibited tumor growth by acting on the HIF-1α/VEGF pathway under hypoxia (111). In addition, the combination of the HIF-1α inhibitor px-478 and the immune-checkpoint inhibitor enhances the cytotoxicity of T cells against tumor cells, which might be related to the blocking of the HIF-1α/LOXL2 signaling pathway (178). Currently, clinical trials of combined therapy with HIF-1α inhibitors are underway. Thus, HIF-1α inhibitors seem to be a promising cancer therapy in the future.

Of note, the selective is not negligible for drug development, anoxic prodrug is an inactive compound that could be activated automatically in a specific anoxic region. This exploits the selective metabolism of precursor drugs in an anoxic environment, diffuses the killing compounds to the whole TME, and realizes the selective killing of tumor cells (179). A randomized controlled trial for the treatment of advanced pancreatic cancer showed that hypoxia-activated prodrug TH-302 combined with GissiTabine drug yields promising results; the combination group achieved more median progression-free survival than the single Gissi treatment group (180). In addition, TH-302 combined immune checkpoint blocking therapy cured >80% of the tumors in a mouse model of prostate cancer, which prolongs the suppression of bone MDSCs and relieves the inhibition of T cell proliferation (181). Another study showed that CP-506, an anoxic prodrug of nitrogen mustard, also yielded satisfactory effects in tumor tissue (182).

Hyperbaric medicine improves hypoxia in the TME by increasing the amount of dissolved oxygen in the blood (183). In previous studies, hyperbaric medicine has shown a satisfactory excellent curative effect in some cancers (breast and ovarian cancer) (184, 185). Hyperbaric oxygen (HBO) can be used as an adjuvant therapy to inhibit tumors by improving the hypoxic microenvironment (186). A recent study showed that hyperbaric medicine in mice with lung cancer improves the anoxic state of tumors, promotes tumor cell apoptosis, and inhibits tumor growth (187).

## Conclusion

Hypoxia is the key factor regulating TME, which mediates the occurrence, development, and drug resistance of tumor cells. Under the condition of hypoxia, TME show immunosuppression and metabolic reprogramming. Therefore, the proliferation and differentiation of immune cells were inhibited. Immunosuppressive cells such as MDSCs, TAM and Tregs are recruited to the hypoxic zone to promote the escape of tumor cells. The metabolic reprogramming of tumor cells is conducive to obtaining energy in a hypoxic environment while maintaining an acidic microenvironment. In addition, glutamine metabolism and fatty acid metabolism have made great contributions to the balanced redox, anti-apoptosis, growth promotion and drug resistance of tumor cells. More importantly, while the hypoxia inhibits the function of PHD, HIF-1α will be activated and promotes the expression of downstream target genes, which further promotes the formation of hypoxic microenvironment and the progress of tumor cells. Therefore, anti-tumor therapy targeting hypoxia and related factors has attracted many researchers’ exploration. Finally, drug resistance induced by hypoxia still plays an important role in the process of anti-tumor treatment, which significantly affects the outcome of treatment. In this review, we summarize the treatment schemes for hypoxia, such as glycolysis inhibitors, anti-angiogenesis drugs, HIF inhibitors, hypoxia-activated prodrugs, and hyperbaric medicine, and finally the found targets and signal pathways in the form of table.

In conclusion, hypoxia is still the key to fight against malignant tumors. It is very necessary to clarify the molecular mechanism of hypoxia on the formation of tumor microenvironment and drug resistance, which will contribute to the breakthrough of tumor targeted therapy in the following work.

## Author contributions

(I) Conception and design: TH, DX. (II) Searching literature and led the writing of the review: DX, GC. (III) Making figures and table: GC, KW, HL. (IV) Final approval of manuscript: All authors.

## Funding

This work was supported in part by the Shanghai “Rising Stars of Medical Talent” Youth Development Program, Outstanding Youth Medical Talents.

## Conflict of interest

The authors declare that the research was conducted in the absence of any commercial or financial relationships that could be construed as a potential conflict of interest.

## Publisher’s note

All claims expressed in this article are solely those of the authors and do not necessarily represent those of their affiliated organizations, or those of the publisher, the editors and the reviewers. Any product that may be evaluated in this article, or claim that may be made by its manufacturer, is not guaranteed or endorsed by the publisher.

## Abbreviations

TME, tumor microenvironment; NK cells, natural killer cells; TAMs, tumor-associated macrophages; IFN-γ, interferon-gamma; DCs, dendritic cells; MHC, Major histocompatibility complex; IL-6, interleukin-6; IL-10, interleukin-10; VEGF, vascular endothelial growth factor; HIF-1α, hypoxia-inducible factor-alpha; BNIP3, BCL2 and adenovirus E1B19 kDa interacting protein 3; PI3K, phosphatidylinositol 3 kinase; MMP10, metalloproteinase 10; VISTA, V-domain Ig suppressor of T cell activation; HLA-G, Human leukocyte antigen G; CAFs, Cancer-associated fibroblasts; TGF-β, tumor growth factor-beta; MDSCs, marrow-derived suppressor cells; Tregs, regulatory T cells; Foxp3, forkhead box P3; ROS, reactive oxygen species; HIFs, Hypoxia-Inducible Factors; PHD, prolyl hydroxylase; HRE, hypoxia response element; P-gp, p-glycoprotein; ETC, electron transport chain; HBO, Hyperbaric oxygen; HK2, Hexokinase 2; 3-BP, 3-bromopyruvic acid; CDT, chemical dynamic therapy; LDHA, lactate dehydrogenase A; αKGDH, α- Ketoglutarate dehydrogenase; ctDNA, circulating tumor DNA; PET-CT, positron emission tomography and computed tomography; ADC, antibody drug conjugate; NSCLC, non-small cell lung cancer.
